# Drug‐related problems among hospitalized hypertensive and heart failure patients and physician acceptance of pharmacists' interventions at a teaching hospital in Ghana

**DOI:** 10.1002/hsr2.786

**Published:** 2022-08-24

**Authors:** Mark Amankwa Harrison, Afia F. A. Marfo, Kwame O. Buabeng, Florence A. Nkansah, Dorcas P. Boateng, Daniel N. A. Ankrah

**Affiliations:** ^1^ Pharmacy Department Korle Bu Teaching Hospital Accra Ghana; ^2^ Department of Pharmacy Practice, Faculty of Pharmacy and Pharmaceutical Sciences, College of Health Sciences Kwame Nkrumah University of Science and Technology Kumasi Ghana

**Keywords:** drug‐related problems, Ghana, heart failure patient, hospitalized patient, hypertensive patient, intervention acceptance

## Abstract

**Background:**

Hypertensive and heart failure patients frequently require multiple drug therapy which may be associated with drug‐related problems (DRPs).

**Aim:**

To determine the frequency, types, and predictors of DRPs, and acceptance of pharmacists' interventions among hospitalized hypertensive and heart failure patients.

**Method:**

It was a prospective cross‐sectional study at the internal medicine department wards of Korle Bu Teaching Hospital (KBTH) between January and June 2019 using a validated form (the pharmaceutical care form used by clinical pharmacists at the medical department). DRPs were classified based on the Pharmaceutical Care Network Europe (PCNE) Classification scheme for DRPs V8.02. Descriptive and inferential statistics were used for data analysis.

**Results:**

A total of 247 DRPs were identified in 134 patients. The mean number of DRPs was 1.84 (SD: 1.039) per patient. Most DRPs occurred during the prescribing process (40.5%; *n*(DRPs) = 100), and the highest prescribing problem was untreated indication (11.7%; *n* = 29). Other frequent DRPs were medication counseling need (25.1%; *n* = 62), administration errors 10.1%(*n* = 25), drug interaction (10.5%; *n* = 26), and “no” or inappropriate monitoring (10.5%; *n* = 26). The number of drugs received significantly predicted the number of DRPs (adjusted odds ratio [AOR]: 9.85; 95% CI: 2.04–47.50; *p* < 0.001). Clinical variables were significant predictors of number of DRPs (diabetic status: AOR: 0.41, 95% CI: 0.18–0.98, *p* < 0.05; statin use: AOR: 0.34, 95% CI: 0.14–0.81, *p* < 0.05; antiplatelet use: AOR: 5.95, 95% CI: 2.03–17.48, *p* < 0.01). Average acceptance of interventions by physicians was 71.6% (SD: 11.7). Most (70.6%; *n* = 48) accepted interventions were implemented by physicians (resolved).

**Conclusion:**

DRPs frequently occur, with most problems identified in the prescribing process. Medication counseling was frequently needed. Patients' number of drugs and clinical factors predicted the occurrence of DRPs. Physicians accepted and implemented most interventions. Our findings suggest that clinical pharmacists have an important role in cardiovascular patient care, but this study should be replicated in other hospitals in Ghana to corroborate these findings.

## 1 INTRODUCTION

Hypertension is increasingly becoming an important public health problem in Sub‐Saharan Africa.^1^, ^2^, ^3^ The disease is an important risk factor for heart failure, which is more prevalent in low socioeconomic settings and the Black population.^4^, ^5^, ^6^, ^7^ Hypertension is the predominant risk factor for heart failure in resource‐limited settings such as Africa.^8^ In Ghana, heart failure is a common reason for hospital attendance and admission among cardiovascular disease patients, and many heart failure patients present with hypertension which is a prevalent health problem in the country.^3^, ^9^, ^10^ Treatment for hypertension and heart failure often requires the use of multiple cardiovascular drugs.^11^, ^12^ Medication use is potentially associated with problems (drug‐related problems [DRPs]) that could affect treatment outcomes, especially when multiple pharmacological agents are required. The problems include medication errors and adverse drug reactions, and they can be classified with validated tools such as the Pharmaceutical Care Network Europe (PCNE), Cipolle's, and APS‐Doc classification systems.^13^, ^14^, ^15^

DRPs lead to substantial morbidity and mortality in addition to cost to health systems. DRPs reportedly caused over 218,000 deaths in the United States in 2000 alone.^16^ The costs resulting from DRPs was estimated to be in excess of 177 billion U.S dollars in the same year.^16^ DRPs have been associated with emergency department visits and hospitalization at internal medicine departments.^17^, ^18^ DRPs arise from prescribing, dispensing, and administration of medication.^19^, ^20^, ^21^, ^22^

The prevalence of DRPs in hospitalized patients varies^23^, ^24^, ^25^ although several studies have shown a common occurrence.^25^, ^26^, ^27^, ^28^ Several studies have reported more than one DRP per patient.^25^, ^27^, ^28^, ^29^, ^30^ Studies have shown variations in DRPs between clinical departments.^25^, ^26^ A study in a tertiary care setting found DRPs to be more common in medical wards than other wards. Two studies showed an average DRP of 2.3 per patient in medical patients^25^, ^31^ while another study found a DRP rate of 1.4 in a tertiary hospital.^32^ The definition of DRPs used by authors also accounts for variations in the reported rates of the problem. Compared with recent studies, an earlier review of DRPs assessing only medication errors found a lower rate of DRPs in hospitalized patients.^23^ Although a review reported medication administration errors as the most prevalent DRPs,^23^ other authors have reported different findings. Prescribing errors have also been reported as the most common DRPs,^20^, ^27^, ^28^ while the occurrence of adverse drug reactions account for a variable proportion.^20^, ^23^, ^24^ DRPs also vary among drugs, geography, and clinical departments/practice of hospitals.^33^ Tigabu et al. concluded that DRPs are common on medical wards.^26^ Although antibiotics have been found to be frequently implicated in DRPs,^20^ cardiovascular drugs including blood pressure lowering medicines, statins, and antithrombotics have also shown a potential to frequently account for DRPs.^19^, ^23^, ^27^, ^34^ Furthermore, the number of drugs prescribed, demographic factors such as age and sex, drugs with a narrow therapeutic index, or renal elimination are likely to influence the frequency of DRPs.^19^, ^23^, ^25^, ^34^

Although DRPs are likely to occur in cardiovascular disease patients,^27^, ^29^, ^35^ the frequency and type of DRPs are likely to vary depending on the method used to detect errors or ADRs (systematic screening of patients vs. chart review or spontaneous reporting) and the clinical departments or wards where studies are conducted.^23^, ^25^ The definitions or classification systems used by authors may also affect DRP findings. Preferable classifications systems for DRPs should have a clear definition, published validation, easy usability in practice, and grouped into main and subgroups (hierarchy of problems).^13^ Despite DRPs being frequently reported, the majority can be prevented, especially through pharmaceutical care activities of pharmacists including monitoring, counseling, and interventions.^19^, ^23^, ^24^, ^25^, ^36^

Acceptance of pharmacists interventions by physicians in hospitalized patients vary although the majority of interventions may be accepted.^31^, ^35^, ^37^, ^38^ A retrospective study of DRPs in hospitalized patients found a rate of intervention acceptance by physicians in 71% of interventions made.^37^ A prospective study reported intervention acceptance of more than 90% by physicians.^31^ Some studies in hospitalized medical patients have, however, reported lower acceptance of pharmacists interventions.^35^, ^38^ Studies in cardiovascular patients have reported varying acceptance rates of pharmacists interventions with some studies reporting acceptance rates of half to two‐thirds of interventions.^35^, ^38^

Most studies on DRPs were conducted in developed countries, leaving developing settings with the paucity of evidence.^20^ In Ghana, evidence on DRPs published in peer‐reviewed journals is very limited. To the knowledge of the authors of this study, this is the first published research evaluating DRPs and their associated factors as well as intervention acceptance in hospitalized hypertensive and heart failure patients in Ghana using a systematic prospective design and the PCNE classification system. The aim of this study was to determine the frequency, types, and predictors of DRPs, and acceptance of pharmacists' interventions among hypertensive and heart failure patients admitted to the internal medicine wards of the Korle Bu Teaching Hospital.

## 2 RESEARCH DESIGN AND METHODS

### 2.1 Study design

It was a prospective cross‐sectional study using a validated data collection form (the pharmaceutical care form used by clinical pharmacists at the medical department of Korle Bu Teaching Hospital) to document patient sociodemographic and clinical information, as well as DRPs and pharmacists' interventions after review by clinical pharmacists. DRPs were classified based on the PCNE Classification scheme for Drug‐Related Problems V8.02.^13^

### 2.2 Study settings

The study was conducted at the department of medicine of the Korle‐Bu Teaching Hospital, Ghana's largest referral hospital which has a bed capacity of 2000. There are 17 clinical and diagnostic departments/Units. It has an average daily attendance of 1500 patients and about 250 patient admissions. The department of medicine had 204 beds at the time of the study, and its subspecialties include gastro‐enterology, Neurology, Rheumatology, Nephrology, Endocrinology, Cardiology, Respiratory, Infectious diseases, and Dermatology.

The main medical block has four floors namely medical 1, 2, 3, and 4 where its wards are located. The medical block had 122 beds at the time of the study, and this number excludes the medical intensive care unit (ICU). These wards admit an average of 200 new patients per month. The medical ICU is located on the ground floor of the medical block. The department has 12 clinical pharmacists.

The main medical block has about 200 nurses, 130 doctors, and 10 clinical pharmacists.

### 2.3 Study participants

The study included hypertensive and heart failure patients admitted to medical wards of the main block of the medical department reviewed and identified by trained clinical pharmacists to have DRPs.

### 2.4 Inclusion criteria

Patients admitted to the four (4) wards during the study period who were 18 years and above were included.

### 2.5 Exclusion criteria

Patients admitted to the medical ICU; patients readmitted to the four wards whose 1st admission was within the study period and all those who were less than 18 years of age were excluded. Patients admitted to satellite wards such as fevers, chest, and stroke units were also excluded.

### 2.6 Sample size calculation and sampling

Sample size for the study was calculated using open source epidermiologic statistics for public health (OpenEpi) for finite populations.^39^ Our calculation was based on a study of cardiovascular admission trends in a tertiary hospital in Ghana over 12 years showing an average annual admission of 159 patients for heart failure with hypertension underlying most admissions.^9^ We used a 39% prevalence of DRPs reported from a study of DRPs in hospitalized cardiovascular patients at a tertiary hospital.^35^ The sample size was obtained using a Confidence level of 95% and confidence limit of 5% was 112. Using systematic sampling every 3rd heart failure and hypertensive patient who met the inclusion criteria was included. We included a total of 134 patients.

Study participants were recruited between January and June 2019.

### 2.7 The PCNE classification system

The PCNE classification system is a validated tool for studying the nature, prevalence, and incidence of DRPs.^13^ It is hierarchical. Unlike other classification systems, problems are separated from the causes (also called medication errors). The PCNE version V8.02 was used for this study. The basic classification has three primary domains for problems, eight primary domains for causes, and five primary domains for Interventions. The system includes classification for “Acceptance of the Intervention Proposals” On a more detailed level, there are seven grouped subdomains for problems, 35 for causes and 16 for interventions, and 10 for intervention acceptance. Subdomains explain the principal domains. It also has a scale to measure the extent of problem resolution.

### 2.8 Data collection

Data were collected using the Korle Bu Teaching Hospital pharmaceutical care form used by clinical pharmacists on medical wards. Before data collection, the care form was validated by testing for face and content validity as well as intra‐ and inter‐rater reliability, and reviewing to ensure validity and reliability. Clinical pharmacists were trained by the investigators on data collection using an illustrative manual.

During the study period, data collectors (clinical pharmacists) obtained the admissions and discharge book of the respective wards to identify all patients admitted for hypertension or heart failure. The medical notes, investigation/laboratory results, vital sign charts, and treatment charts of these patients were reviewed by the clinical pharmacists (data collector) to identify and document DRPs (pharmaceutical care issues). Interventions were verbally communicated to physicians through face‐to‐face meetings and phone calls. The clinical pharmacist tracked the status of any interventions proposed by monitoring daily patient reviews in the medical notes as well as treatment charts. Using the pharmaceutical care form, the clinical pharmacist documented the patient's demographic and clinical data, DRPs, interventions, acceptance, and implementation of the intervention. The investigators then retrieved the completed pharmaceutical care forms and made a list of folder numbers of patients with DRPs for sampling and inclusion. Simple random sampling with Microsoft Excel was then used to select among the first three patients on the list to include the first patient. Using systematic sampling, every 3rd patient with DRP was subsequently included. This process was repeated for subsequent admissions until 134 hypertensive and heart failure patients were included. DRPs, interventions, and their acceptance were classified using the PCNE classification system version V8.02.

Independent variables collected were sociodemographic and clinical characteristics. The key dependent variable collected was the frequency of DRPs. The key independent variables collected were the number of drugs and clinical factors. Other variables collected were demographic characteristics, categories of DRPs, and pharmacists' interventions (including their acceptance and implementation).

Consent was sought from patients whose cases were included in the study after data collectors explained the study to them.

### 2.9 Outcome measures

Types/categories of DRPs, most frequent DRPs, frequency of DRPs per patient, interventions for DRPs, acceptance of pharmacists' interventions, and implementation of accepted interventions

*Types/categories of DRPs*: DRPs were classified into categories using the PCNE classification system version V8.02.

The primary domains of DRPs classified under prescribing problems (errors) were drug selection, drug form, dose selection, and treatment duration. Problems of the drug use process, patient‐related problems and other problems such as inappropriate monitoring and interaction were classified under drug use problems.

Frequency of DRPs was determined by calculating the average number of DRPs per patient and proportion of each DRP. Interactions were checked using Medscape reference.

*Interventions*: These were classified using the PCNE version V8.02.^13^

*Frequency of interventions*: this was determined by calculating the average number of interventions per patient.

Acceptance of interventions by physicians was measured as an average percentage of interventions made at the physicians' level accepted by physicians. The outcome of interventions accepted by physicians was classified as resolved, unresolved or status unknown and a proportion of each of the three outcomes was determined.

Definitions used for the various categories/types of DRPs in this study can be found in Supporting Information: Appendix 1.

### 2.10 Data analysis

Data were analyzed using the statistical package for social scientist, SPSS version 22. Univariate, bivariate, and multivariate analysis were performed. Categorical variables were expressed as frequencies and percentages; while continuous variables were described using means and standard deviation.

*χ* ^2^ tests were used to test the association between categorical variables. Logistic regression was used to determine the relationship between dependent and independent variables, and to determine the predictors of DRPs. *p* < 0.05 was considered statistically significant.

### 2.11 Ethical approval

The study was approved by the institutional review board of the Korle Bu Teaching Hospital.

## 3 RESULTS

### 3.1 Demographic characteristics

Data collection forms were completed for 134 patients (hypertensive (*n*) = 126; heart failure (*n*) = 56). The mean age of the study participants was 55.18 (SD: 16.95), and more than one‐third (38.81%; 52/134) were older than 60 years. Sixty‐eight patients (50.7%; 68/134) were males, and most patients (84.3%; 113/134) were subscribers of the national health insurance scheme (NHIS) (Table 1).

**Table 1:** Demographic and clinical characteristics of hospitalized hypertensive and heart failure study participants (*N* = 134) at the Korle Bu teaching hospital in 2019

Characteristic	Mean	SD
Age (mean)	55.18	16.95
Less than 40 years	26	19.40
40 – 60 years	56	41.79
Above 60 years	52	38.81

**Characteristic**	**Frequency**	**%**
Sex		
Male	68	50.7
female	66	49.3
National health insurance (NHIS) status		
Yes	113	84.3
No	21	15.7

**Clinical characteristic**	**Frequency**	**Percentage**
Type of ward		
Cardiology/Gastroenterology	96	71.6
Neuroendocrine	8	6
Respiratory/Rheumatology	23	17.2
Dermatology	7	5.2
Diabetic	28	20.9
Hypertensive	126	94
Heart failure patients	56	41.8

**Clinical characteristic**	**Average frequency (mean)**	**SD**
Number of comorbidities	2.65	1.3
Number of drugs(mean)	8.92	3.2
Number of blood pressure drugs (hypertensive patients)	2.77	1.7
Number of heart failure drugs (heart failure patients)	2.77	1.15
Number of parenteral drugs	2.89	1.56

### 3.2 Clinical characteristics of study participants

The average number of comorbidities per patient was 2.65 (SD: 1.3), and 28 patients (20.9%; 28/134) were diabetic. The average number of drugs per patient was 8.92 (SD: 3.2). The average number of parenteral drugs was 2.89 (SD: 1.57). One hundred and twenty‐six patients were hypertensive and 56 had heart failure. Details of clinical characteristics can be found in Table 1.

### 3.3 DRPs

A total of 247 DRPs were identified. The mean number of DRPs was 1.84 (SD: 1.039) per patient (Table 2). About 20% of patients (27/134) had three or more DRPs. Most DRPs (98.4%; 243/247) were medication errors. Most of them (DRP) occurred during the prescribing process (40.5%; 100/247). The frequency of medication administration errors was 10.1% (25/247). The most common prescribing error was an untreated indication (no drug treatment for existing indication) (11.7%; 29/247). Medication counseling need (25.1%; 62/247) was more frequent than any individual DRP. This was followed by No drug treatment for existing indication (11.7%; 29/47), drug interaction (10.5%; 26/247), no or inappropriate monitoring (10.5%; 26/247), drug under‐administered or not administered at all (9.3%; 23/247), and so on. Four DRP (1.6%; 4/247) were suspected adverse drug reactions. Details of DRPs can be found in Table 2.

**Table 2:** Drug‐related problems identified among hospitalized hypertensive and heart failure study participants (*N* = 134) at the Korle Bu teaching hospital in 2019

Drug‐related problem	Frequency	%
Medication Counseling need No drug treatment for existing indication Drug interaction No or inappropriate monitoring Drug under‐administered or not administered at all (missed doses) Dose too high Inappropriate Drug selection	62 29 26 26 23 22 13	25.1 11.7 10.5 10.5 9.3 8.9 5.3
Duplication of therapy Dose too frequent or not frequent enough Contraindication Adverse drug reaction Inadequate response No indication for drug use Treatment duration too long or short Inappropriate drug form	12 9 5 4 4 3 3 2	4.9 3.6 2.0 1.6 1.6 1.2 1.2 0.8
Drug dose too low	2	0.8
Inappropriate timing and dosing interval of drug administration (admin errors)	2	0.8
Total	247	100

### 3.4 Drugs frequently related to problems

Anticoagulants were mostly implicated in DRPs (32.8%; 64/195). Counseling needs and interactions were the main problems related to anticoagulants. Warfarin was the main anticoagulant related to problems. Blood pressure medication, antibiotics, analgesics, antiplatelets, and antidiabetics were related to 15.9%(31/195), 15.4%(30/195), 8.2%(16/195), 5.1%(10/195), and 5.1%(10/195) of DRPs, respectively. Statins were implicated in six problems (3.1%; 6/195). Details of drugs frequently related to problems are shown in Table 3.

**Table 3:** Drugs frequently related with problems in hospitalized hypertensive and heart failure study participants (*N* = 134) at the Korle Bu teaching hospital in 2019

Drug class	Frequency	%
Antibiotics	30	15.38
Anticoagulants	64	32.82
Analgesics	16	8.21
Antidiabetic	10	5.12
Blood pressure drugs	31	15.9
Statin	6	3.08
Antiplatelet	10	5.12
Antiulcer	9	4.62
Corticosteroids	7	3.59
Antiallergy	3	1.54
DMARD	3	1.54
Haematinics	3	1.54
Cardiac stimulants	3	1.54
Total	195	100

Abbreviation: DMARD, disease‐modifying antirheumatic drug.

### 3.5 Relationship between number of drugs, demographic, and clinical variables and number of DRPs

In a logistic regression analysis, the number of drugs received by patients significantly predicted the number of DRPs (Table 4). Patients who had been on 15 or more drugs were more likely to have three or more DRPs (adjusted odds ratios [AOR]: 9.85; 95% CI: 2.04–47.50; *p* < 0.001). Clinical variables (diabetic status, statin use, and antiplatelet drug use) had a significant relationship with a number of DRPs from AOR. Patients who were not diabetic were less likely than those who were diabetic to have three or more DRPs (AOR: 0.41; 95% CI: 0.18–0.98; *p* < 0.05). Demographic variables did not significantly predict the number of DRPs from adjusted odds ratios. Based on crude odds ratios, those who did not have National Health Insurance were less likely than those with National Health Insurance to have three or more DRPs (crude odds ratios [COR]: 0.13; 95% CI: 0.09‐–0.96; *p* < 0.05) but significance was lost from adjusted odds ratios (AOR: 0.28; 95% CI: 0.03–2.24). From adjusted odds ratios, age did not significantly predict the number of DRPs (AOR: 0.52: 95% CI: 0.17–1.60) although a relationship was found from COR (COR: 0.15; 95% CI: 0.04–0.54; *p* < 0.01). Table 4 shows the adjusted and crude odds ratios for the number of drugs, demographic, and clinical variables, their confidence intervals and *p* values.

**Table 4:** Relationship between number of drugs and number of drug‐related problems while controlling for demographic and clinical factors (odds ratios, their confidence intervals and *p* values) in hospitalized hypertensive and heart failure study participants (*N* = 134) at the Korle Bu teaching hospital in 2019 using logistic regression

Variables	AOR (95% CI)	COR (95% CI)
Number of drugs		
Less than 10 drugs	Reference	Reference
10–14 drugs	1.67 (0.66–4.22)	1.22 (0.42–3.49)
15 and more drugs	9.85*** (2.04–47.50)	8.75 (0.74–104.10)
Age		
Less than 40 years	Reference	Reference
40–60 years	0.52 (0.17–1.60)	0.15** (0.04–0.54)
More than 60 years	0.73 (0.24–2.18)	0.17* (0.04–0.68)
Sex		
Male	Reference	Reference
Female	2.01 (0.84–4.81)	2.03 (0.69–6.01)
National Health Insurance subscription		
Yes	Reference	Reference
No	0.28 (0.03–2.24)	0.13* (0.09–0.96)
Statin		
Yes	Reference	Reference
No	0.34* (0.14–0.81)	0.13 (0.29–3.73)
Diabetes		
Yes	Reference	Reference
No	0.41* (0.18–0.98)	0.23** (0.08–0.67)
Antiplatelet		
0	Reference	Reference
1	3.01 (0.90–10.10)	7.35* (1.34–40.31)
2	5.95** (2.03–17.48)	9.75** (2.01–47.34)

Abbreviations: AOR, adjusted odds ratio; COR, crude odds ratio.

* *p* < 0.05.

** *p* < 0.01.

*** *p* < 0.001.

### 3.6 Acceptance and implementation of pharmacists' interventions by physicians

**Figure 1:**
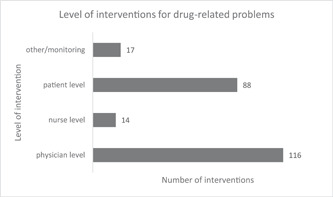
Level of interventions for drug‐related problems among hypertensive and heart failure study participants (*N* = 134) at the Korle Bu teaching hospital in 2019

A total of 235 pharmaceutical care interventions were made by pharmacists to resolve DRPs. The average number of interventions per patient was 1.75 (SD: 1.000). Majority of interventions were made at the physician level (49.4%; 116/235). Eighty‐eight interventions (37.4%; 88/235) were made at the patient level while 14 (6%; 14/235) were made at the nurses level (Figure 1).

The average acceptance of physician‐level interventions by physicians was 71.6% (SD: 11.7). All interventions at the physician level were accepted in 44 patients. Of 15 patients, 50% of interventions were accepted. In 1 and 2 patients 60% and 75% were accepted, respectively. Of 12 patients, no intervention was accepted. In most patients (70.6%; *n* = 48) accepted interventions at the physician level were implemented by physicians (resolved). The status of 17 accepted interventions (25%) was unknown. Three accepted interventions (4.4%) were not resolved.

## 4 DISCUSSION

In this study, DRPs were common in hypertensive and heart failure patients. DRPs occurred more frequently during the prescribing process than at any other stage in patient treatment. The most common prescribing error was an untreated indication. Medication counseling need was common. Anticoagulants were the most implicated medication in DRPs. The number of drugs prescribed for patients was a predictor of DRPs. Pharmaceutical care interventions were frequently proposed by clinical pharmacists. Majority of interventions were proposed at the physician level, and most interventions were accepted by physicians. Majority of interventions accepted by physicians were implemented to solve DRPs.

There were more patients with hypertension than heart failure. This reflects the high prevalence of hypertension in Ghana.^1^, ^40^ The total number of hypertensive and heart failure cases exceeded the total number of patients. This shows that some patients had concomitant hypertension and heart failure which reflects the frequent comorbidity of hypertension and heart failure in medical patients.^9^ In this study, comorbidity was common and one‐fifth of patients were diabetic. Studies show that diabetes is a risk factor for hypertension and heart failure and frequently coexists with these diseases.^9^, ^41^ Several drugs were frequently prescribed (polypharmacy) for patients. Polypharmacy is common in the management of cardiovascular patients.^42^ Hypertensive and heart failure patients require multiple medications for BP control and cardiovascular risk reduction as well as morbidity and mortality reduction in heart failure, respectively.^12^

DRPs were frequently identified during the study. This is consistent with the findings of other studies on cardiovascular and other hospitalized medical patients.^20^, ^25^, ^27^, ^29^, ^31^, ^35^ DRPs have been shown to be frequent in patients with cardiovascular disease.^27^, ^29^, ^31^, ^35^ Movva et al. reported a high occurrence of DRPs in cardiovascular patients in their study which classified DRPs based on the PCNE classification system.^31^ Patients with cardiac and vascular disorders such as hypertension and heart failure frequently have comorbid conditions and require multiple medications for treatment. This is likely to account for the frequent finding of DRPs. Similar to our findings, DRPs have been reported to be common in tertiary hospitals in Africa.^27^, ^30^ Close to two DRPs were found per patient in our study. Studies in hypertensive and heart failure patients as well as those hospitalized for other cardiovascular conditions have also reported more than one DRP per patient.^27^, ^29^, ^31^ Two studies in patients with cardiovascular disease reported two DRPs per patient.^29^, ^31^ Another study by Niriayo et al. in heart failure patients identified more than two DRPs per patient.^27^ The high occurrence of DRPs suggests that the achievement of optimal outcomes in hypertensive and heart failure patients is threatened without the pharmaceutical care input of clinical pharmacists, and further suggests the critical need for clinical pharmacists on the medical wards.

DRPs occurred more frequently during the prescribing process. This finding corroborates reports from several studies, including those in cardiovascular disease patients.^20^, ^26^, ^27^, ^31^, ^43^, ^44^ A review of DRPs in hospitalized patients showed prescribing errors as a common occurrence, which affects 7% of medication orders and 50% of hospital admissions.^20^ However, other studies have reported different findings.^23^, ^32^ A review of 35 studies by Krähenbühl‐Melcher et al. reported medication administration errors as the most frequent DRP in hospitalized patients.^23^ This variation in the commonest DRP reported, most likely stems from the use of varying methods for detecting (systematic screening vs. chart reviews) and for defining and classifying DRPs (hierarchical vs. other methods).^20^, ^23^ Among DRPs that occurred during the prescribing process, untreated indication was the most common. Reports of studies, including systematic reviews in hospitalized patients by other authors, corroborate this finding.^26^, ^43^, ^44^ Gelchu et al., in their study of DRPs in cardiovascular disease patients at a tertiary hospital, identified untreated indications in more than half of DRPs.^43^ Other identified prescribing problems (errors) were dosing problems and inappropriate drug selection. Studies, including those conducted in heart failure and other cardiovascular patients, have shown similar results.^20^, ^27^, ^31^, ^32^

In a quarter of DRPs, the need for medication counseling as a result of nonadherence or the use of drugs with significant interaction with food was identified. Studies of DRPs in cardiovascular patients have shown that nonadherence is a common problem.^27^, ^43^ A study in heart failure patients classifying noncompliance as a DRP at a teaching hospital identified the problem in a quarter of patients, while Gelchu et al. found nonadherence as a DRP in more than 10% of DRPs in patients with cardiovascular disease.^27^, ^43^ A systematic review and meta‐analysis by Adem et al. has linked nonadherence to the occurrence of DRPs.^44^ The frequent use of multiple medications and the resulting cost and need for long‐term use may substantially account for nonadherence in hypertensive and heart failure patients. Drug interactions, inappropriate monitoring, and medication administration errors were each identified in about one‐tenth of DRPs. Several studies have identified drug interactions, administrative errors, and inappropriate monitoring as a common occurrence in cardiovascular and other medical patients.^21^, ^23^, ^25^, ^32^ The need for multiple medications for the treatment of hypertensive and heart failure patients is most likely to increase the risk of drug interactions and administrative errors. Many cardiovascular drugs are associated with biochemical and clinical disturbances, necessitating relevant monitoring of treatment.^45^

Anticoagulants were the drugs most frequently implicated in DRPs. Acheampong et al. have shown similar findings in Ghana.^19^ In a prospective study of heart failure patients at a teaching hospital in Ethiopia, Niriayo et al. reported anticoagulants among the drugs most frequently implicated in DRPs.^27^ A review of DRPs has shown that the use of anticoagulants is associated with a higher occurrence of DRPs.^23^ Anticoagulants are potentially associated with important risks, and oral drugs such as warfarin have a narrow therapeutic index and important potential interactions.^46^ These characteristics as well as the need for their clinical or laboratory monitoring or both and their potential interaction with organ function are likely to account for their frequent association with DRPs. In addition to blood pressure medication, statins and antiplatelets were also implicated in DRPs. Patients with cardiovascular disorders such as hypertension frequently receive statins for primary and secondary prevention of cardiovascular events and antiplatelets for secondary prevention.^12^ The occurrence of statin and antiplatelet‐related DRPs in addition to blood pressure medication‐related DRPs suggests that many hypertensive patients as well as heart failure patients with atherosclerosis are very likely to have DRPs. The frequent occurrence of antibiotic‐related problems in our study is consistent with findings of a review of DRPs in hospitalized patients.^20^ Antimicrobials are frequently used in hospitalized patients.^47^ Cardiovascular patients such as those with heart failure may be hospitalized as a result of precipitating infections such as pneumonia. These probably accounts for the frequent occurrence of antibiotic‐related problems in our study.

The number of drugs received by patients significantly predicted the number of DRPs. This finding is consistent with published findings on DRPs in hospitalized patients from several studies including systematic reviews and meta‐analysis.^23^, ^24^, ^25^, ^26^, ^43^, ^44^ An earlier review and a recent systematic review and meta‐analysis have shown a relationship between polypharmacy and the occurrence of DRPs.^23^, ^44^ Studies in hypertensive and heart failure patients in teaching hospitals have reported a higher risk of DRPs in patients taking a higher number of drugs.^27^, ^43^ Multiple medication use (polypharmacy) increases the complexity of treatment and contributes to drug interactions and medication nonadherence and medication use errors. Multiple medication use also increases the likelihood of medication interaction with disease and organ function. Demographic factors did not significantly predict the number of DRPs when the analysis was adjusted for confounding factors, although a relationship was found from unadjusted analysis. This suggests a probable interaction between demographics and other variables. A study of cardiovascular patients at a teaching hospital in Ethiopia did not find a significant relationship between demographic factors and DRPs.^43^ Some studies have, however, reported a relationship between demographic variables and the number of DRPs.^23^, ^25^, ^26^, ^27^ Variations in analytical methods as well as methods and definitions used for studying DRPs probably account for the disagreements in relationship findings. Clinical factors (diabetic status, statin use, and antiplatelet use) significantly predicted the number of DRPs. This finding has been reported in previous studies.^26^, ^27^, ^44^ Diabetic comorbidity, in addition to morbidity burden, contributes to polypharmacy and increases the risk of DRPs. Statins and antiplatelets have a potentially important risk of toxicity and interaction, and may contribute to adherence problems.

Pharmaceutical care interventions were frequently proposed by pharmacists to resolve DRPs. The majority of interventions were proposed at the physician level. Our findings are similar to those of other authors.^31^, ^35^, ^48^, ^49^, ^50^, ^51^ A prospective study of DRPs in hospitalized patients at a tertiary hospital by George et al. reported prescriber‐level interventions by pharmacists in 41.4% of all interventions, representing the highest percentage.^48^ A higher percentage of interventions (97%) attributed to prescriber‐level interventions was shown in another prospective study in hospitalized patients managed by internal medicine physicians.^49^ The higher percentage of interventions at the prescriber level reflects the higher percentage of DRPs at the prescribing stage of care found in our study. A significant proportion of interventions were made at the patient level. Many patients in our study needed medication counseling due to nonadherence or the use of medication with significant food interaction. Studies in cardiovascular patients have shown that the need for medication counseling of hospitalized patients frequently occurs.^27^, ^43^, ^52^

An average of about 72% of pharmacists' interventions proposed at the physician level were accepted. This finding is similar to findings from the work of other authors. The majority of interventions proposed by pharmacists in the management of medical patients have been shown to be accepted by physicians.^31^, ^37^, ^48^, ^49^, ^50^, ^51^ A retrospective study of DRPs in hospitalized patients found a rate of intervention acceptance by physicians in 71% of interventions made.^37^ Divergent clinical opinions and a lack of clearly documented treatment guidelines may account for physician nonacceptance of pharmacists' interventions. Some prospective studies have reported intervention acceptance of more than 90% by physicians.^31^, ^51^ While interventions may be accepted, their acceptance rate may differ from the implementation rate.^31^ In our study, most interventions accepted by physicians were implemented to resolve DRPs. This finding is comparable to the findings of the work of other authors.^49^, ^52^ The high physician acceptance rate of clinical pharmacists' interventions suggests that clinical pharmacists have a central role in the optimization of pharmacotherapeutic outcomes. The impact of clinical pharmacists' interventions has been studied in hospitalized Patients.^36^, ^53^, ^54^, ^55^ A meta‐analysis and systematic review of randomized controlled trials of pharmacists interventions in heart failure patients has shown that pharmacists interventions reduce all‐cause mortality and hospitalization in heart failure.^55^ A review of 36 studies in hospitalized patients evaluating pharmacists' participation in ward rounds, medication reconciliation, and drug‐specific services also showed better treatment outcomes.^36^ The review showed a reduction in medication errors and adverse drug events, as well as better medication adherence and shortened hospital stays.^36^

### 4.1 Limitations of the study

The study did not include patients on satellite wards and the intensive care unit of the medical department of the hospital. The relationship between renal and liver function and DRPs was not evaluated. The study was conducted at a single site. It may be challenging to extend the findings to other hospitals.

## 5 CONCLUSION

DRPs frequently occur in hypertensive and heart failure patients with most problems identified in the prescribing process. Medication counseling for patients was frequently needed. The number of drugs received by patients and clinical factors predicted the occurrence of DRPs. Physicians accepted and implemented most interventions proposed by clinical pharmacists. This may have improved outcomes. Our findings suggest that clinical pharmacists have an important role in cardiovascular patient care. This study should be replicated in other hospitals in Ghana. The influence of interventions on clinical and economic outcomes should also be evaluated in future studies.

## AUTHOR CONTRIBUTIONS

Mark Amankwa Harrison, Daniel N. A. Ankrah, Afia F. A. Marfo and Kwame O. Buabeng contributed to conception and design of the study, as well as analysis and interpretation of data. Florence A. Nkansah contributed to data interpretation. Mark Amankwa Harrison, Afia F. A. Marfo, Kwame O. Buabeng, Florence A. Nkansah, Daniel N. A. Ankrah, and Dorcas P. Boateng contributed to drafting the article and revising it critically for important intellectual content. All authors of this manuscript gave final approval to the version to be published.

## CONFLICT OF INTEREST

The authors declare no conflict of interest.

## TRANSPARENCY STATEMENT

The lead author [Mark Amankwa Harrison] affirms that this manuscript is an honest, accurate, and transparent account of the study being reported; that no important aspects of the study have been omitted; and that any discrepancies from the study as planned (and, if relevant, registered) have been explained.

## Supporting information

Supporting information.

## Data Availability

The data used to support the findings of this study are available from the corresponding author upon reasonable request.

## References

[1] Bosu WK , Reilly ST , Aheto JMK , Zucchelli E . Hypertension in older adults in Africa: a systematic review and meta‐analysis. PLoS One. 2019;14(4):e0214934. 30951534 10.1371/journal.pone.0214934 PMC6450645

[2] Guwatudde D , Nankya‐Mutyoba J , Kalyesubula R , et al. The burden of hypertension in sub‐saharan Africa: a four‐country cross sectional study. BMC Public Health. 2015;15(1):1‐8. 26637309 10.1186/s12889-015-2546-z PMC4670543

[3] Addo J , Smeeth L , Leon DA . Hypertension in sub‐saharan Africa: a systematic review. Hypertension. 2007;50(6):1012‐1018. 17954720 10.1161/HYPERTENSIONAHA.107.093336

[4] Dunlay SM , Weston SA , Jacobsen SJ , Roger VL . Risk factors for heart failure: a population‐based case‐control study. Am J Med. 2009;122(11):1023‐1028. 19854330 10.1016/j.amjmed.2009.04.022 PMC2789475

[5] Levy D , Larson MG , Vasan RS , Kannel WB , Ho KK . The progression from hypertension to congestive heart failure. JAMA. 1996;275(20):1557‐1562. 8622246

[6] Bahrami H . Risk factors for incident congestive heart failure: insights from the multi‐ethnic study of atherosclerosis. The Johns Hopkins University; 2008.

[7] Hawkins NM , Jhund PS , McMurray JJ , Capewell S . Heart failure and socioeconomic status: accumulating evidence of inequality. Eur J Heart Fail. 2012;14(2):138‐146. 22253454 10.1093/eurjhf/hfr168

[8] Vos T , Flaxman AD , Naghavi M , et al. Years lived with disability (YLDs) for 1160 sequelae of 289 diseases and injuries 1990–2010: a systematic analysis for the global burden of disease study 2010. Lancet. 2012;380(9859):2163‐2196. 23245607 10.1016/S0140-6736(12)61729-2 PMC6350784

[9] Appiah LT , Sarfo FS , Agyemang C , et al. Current trends in admissions and outcomes of cardiac diseases in Ghana. Clin Cardiol. 2017;40(10):783‐788. 28692760 10.1002/clc.22753 PMC6490361

[10] Owusu IK , Boakye YA . Prevalence and aetiology of heart failure in patients seen at a teaching hospital in Ghana. J Cardiovasc Dis Diagn. 2013;1(131):2.

[11] Ponikowski P , Voors AA , Anker SD , et al. 2016 ESC guidelines for the diagnosis and treatment of acute and chronic heart failure. Kardiol Pol. 2016;74(10):1037‐1147. 27748494 10.5603/KP.2016.0141

[12] Williams B , Mancia G , Spiering W , et al. 2018 ESC/ESH guidelines for the management of arterial hypertension: the Task Force for the management of arterial hypertension of the European Society of Cardiology (ESC) and the European Society of hypertension (ESH). Eur Heart J. 2018;39(33):3021‐3104. 30165516 10.1093/eurheartj/ehy339

[13] Europe Pharmaceutical Care Network . Foundation. The Pharmaceutical Care Network Europe Classification for drug related problems V8.02. PCNE Foundation. 2017. Available from: https://www.pcne.org/upload/files/300_PCNE_classification_V8-02n.pdf

[14] Cipolle RJ , Strand LM , Morley PC . Pharmaceutical care practice: the patient‐centered approach to medication management. McGraw Hill Professional; 2012.

[15] Hohmann C , Eickhoff C , Klotz JM , Schulz M , Radziwill R . Development of a classification system for drug‐related problems in the hospital setting (APS‐Doc) and assessment of the inter‐rater reliability. J Clin Pharm Ther. 2012;37(3):276‐281. 21790687 10.1111/j.1365-2710.2011.01281.x

[16] Ernst FR , Grizzle AJ . Drug‐related morbidity and mortality: updating the cost‐of‐illness model. J Am Pharm Assoc. 2001;41(2):192‐199. 10.1016/s1086-5802(16)31229-3 11297331

[17] Patel P , Zed PJ . Drug‐related visits to the emergency department: how big is the problem? Pharmacotherapy. 2002;22(7):915‐923. 12126224 10.1592/phco.22.11.915.33630

[18] Samoy LJ , Zed PJ , Wilbur K , Balen RM , Abu‐Laban RB , Roberts M . Drug‐related hospitalizations in a tertiary care internal medicine service of a Canadian hospital: a prospective study. Pharmacotherapy. 2006;26(11):1578‐1586. 17064202 10.1592/phco.26.11.1578

[19] Acheampong F , Nkansah FA , Anto BP . Drug‐related problems and their clinical interventions in a Ghanaian teaching hospital. Saf Health. 2016;2(1):1‐7.

[20] Lewis PJ , Dornan T , Taylor D , Tully MP , Wass V , Ashcroft DM . Prevalence, incidence and nature of prescribing errors in hospital inpatients. Drug Saf. 2009;32(5):379‐389. 19419233 10.2165/00002018-200932050-00002

[21] Avery AJ , Ghaleb M , Barber N , et al. The prevalence and nature of prescribing and monitoring errors in English general practice: a retrospective case note review. Br J Gen Pract. 2013;63(613):e543‐e553. 23972195 10.3399/bjgp13X670679 PMC3722831

[22] James KL , Barlow D , McArtney R , Hiom S , Roberts D , Whittlesea C . Incidence, type and causes of dispensing errors: a review of the literature. Int J Pharm Pract. 2009;17(1):9‐30. 20218026

[23] Krähenbühl‐Melcher A , Schlienger R , Lampert M , Haschke M , Drewe J , Krähenbühl S . Drug‐related problems in hospitals. Drug Saf. 2007;30(5):379‐407. 17472418 10.2165/00002018-200730050-00003

[24] Dequito AB , Mol PG , van Doormaal JE , et al. Preventable and non‐preventable adverse drug events in hospitalized patients. Drug Saf. 2011;34(11):1089‐1100. 21981436 10.2165/11592030-000000000-00000

[25] Lenssen R , Heidenreich A , Schulz JB , et al. Analysis of drug‐related problems in three departments of a German university hospital. Int J Clin Pharm. 2016;38(1):119‐126. 26511945 10.1007/s11096-015-0213-1

[26] Tigabu BM , Daba D , Habte B . Drug‐related problems among medical ward patients in Jimma university specialized hospital, southwest Ethiopia. J Res Pharm Pract. 2014;3(1):1‐5. 24991628 10.4103/2279-042X.132702 PMC4078648

[27] Niriayo YL , Kumela K , Kassa TD , Angamo MT . Drug therapy problems and contributing factors in the management of heart failure patients in jimma university specialized hospital, southwest Ethiopia. PLoS One. 2018;13(10):e0206120. 30352096 10.1371/journal.pone.0206120 PMC6198973

[28] Garedow AW , Mulisa Bobasa E , Desalegn Wolide A , et al. Drug‐related problems and associated factors among patients admitted with chronic kidney disease at jimma university medical center, jimma zone, jimma, southwest Ethiopia: a hospital‐based prospective observational study. Int J Nephrol. 2019;2019:1504371. 10.1155/2019/1504371 31772774 PMC6854244

[29] Redzuan AM , Ramli AR , Pheng MTH . Drug‐related problems in hypertensive patients with multiple comorbidities. J Pharm Res. 2017;1(3):000113.

[30] Yimama M , Jarso H , Desse TA . Determinants of drug‐related problems among ambulatory type 2 diabetes patients with hypertension comorbidity in southwest Ethiopia: a prospective cross sectional study. BMC Res Notes. 2018;11(1):1‐6. 30249291 10.1186/s13104-018-3785-8 PMC6154819

[31] Movva R , Jampani A , Nathani J , Pinnamaneni SH , Challa SR . A prospective study of incidence of medication‐related problems in general medicine ward of a tertiary care hospital. J Adv Pharm Technol Res. 2015;6(4):190‐194. 26605161 10.4103/2231-4040.166502 PMC4630727

[32] Sarfaraz M , Mathew B , Poudel S . Assessment of drug‐related problems in a tertiary care teaching hospital, India. Asian J Pharm Clin Res. 2017;10:310‐313.

[33] van den Bemt PM , Egberts TC , de Jong‐van den Berg LT , Brouwers JR . Drug‐related problems in hospitalised patients. Drug Saf. 2000;22(4):321‐333. 10789826 10.2165/00002018-200022040-00005

[34] Wilmer CM , Huiskes VJB , Natsch S , Rennings AJM , van den Bemt BJF , Bos JM . Drug‐related problems in a clinical setting: a literature review and cross‐sectional study evaluating factors to identify patients at risk. Eur J Hosp Pharm. 2015;22(4):229‐235.

[35] Shareef J , Sandeep B , Shastry CS . Assessment of drug related problems in patients with cardiovascular diseases in a tertiary care teaching hospital. J Pharm Care . 2014;2(2):70‐76.

[36] Kaboli PJ , Hoth AB , McClimon BJ , Schnipper JL . Clinical pharmacists and inpatient medical care: a systematic review. Arch Intern Med. 2006;166(9):955‐964. 16682568 10.1001/archinte.166.9.955

[37] Zaal RJ , den Haak EW , Andrinopoulou ER , van Gelder T , Vulto AG , van den Bemt P . Physicians' acceptance of pharmacists' interventions in daily hospital practice. Int J Clin Pharm. 2020;42(1):141‐149. 32026348 10.1007/s11096-020-00970-0 PMC7162822

[38] Laranjeira T , Mirco A , Falcão F 4CPS‐027 Hospital pharmacist interventions in an accredited cardiology department. 2018.

[39] Sullivan KM , Dean A , Soe MM . On academics: OpenEpi: a web‐based epidemiologic and statistical calculator for public health. Public Health Rep. 2009;124(3):471‐474. 19445426 10.1177/003335490912400320 PMC2663701

[40] Addo J , Agyemang C , Smeeth L , de‐Graft Aikins A , Edusei AK , Ogedegbe O . A review of population‐based studies on hypertension in Ghana. Ghana Med J. 2012;46(2):4‐11. 23661811 PMC3645150

[41] Tsimihodimos V , Gonzalez‐Villalpando C , Meigs JB , Ferrannini E . Hypertension and diabetes mellitus: coprediction and time trajectories. Hypertension. 2018;71(3):422‐428. 29335249 10.1161/HYPERTENSIONAHA.117.10546 PMC5877818

[42] Tefera YG , Alemayehu M , Mekonnen GB . Prevalence and determinants of polypharmacy in cardiovascular patients attending outpatient clinic in Ethiopia university hospital. PLoS One. 2020;15(6):e0234000. 32479516 10.1371/journal.pone.0234000 PMC7263581

[43] Gelchu T , Abdela J . Drug therapy problems among patients with cardiovascular disease admitted to the medical ward and had a follow‐up at the ambulatory clinic of Hiwot fana specialized university hospital: the case of a tertiary hospital in eastern Ethiopia. SAGE Open Med. 2019;7:2050312119860401. 31367379 10.1177/2050312119860401 PMC6643177

[44] Adem F , Abdela J , Edessa D , Hagos B , Nigussie A , Mohammed MA . Drug‐related problems and associated factors in Ethiopia: a systematic review and meta‐analysis. J Pharm Policy Pract. 2021;14(1):1‐24. 33902729 10.1186/s40545-021-00312-z PMC8077957

[45] Whelton PK , Carey RM , Aronow WS , et al. 2017 ACC/AHA/AAPA/ABC/ACPM/AGS/APhA/ASH/ASPC/NMA/PCNA guideline for the prevention, detection, evaluation, and management of high blood pressure in adults: a report of the American college of Cardiology/American Heart Association Task Force on clinical practice guidelines. J Am Coll Cardiol. 2018;71(19):e127‐e248. 29146535 10.1016/j.jacc.2017.11.006

[46] Steffel J , Verhamme P , Potpara TS , et al. The 2018 European Heart Rhythm Association practical guide on the use of non‐vitamin K antagonist oral anticoagulants in patients with atrial fibrillation. Eur Heart J. 2018;201839(16):1330‐1393. 10.1093/eurheartj/ehy136 29562325

[47] Versporten A , Zarb P , Caniaux I , et al. Antimicrobial consumption and resistance in adult hospital inpatients in 53 countries: results of an Internet‐based global point prevalence survey. Lancet Glob Health. 2018;6(6):e619‐e629. 29681513 10.1016/S2214-109X(18)30186-4

[48] George RM , James E , Vijayalakshmi S . Clinical pharmacist's interventions on drug related problems in a tertiary care hospital. Int J Pharm Pharm Sci. 2015;7(6):401‐404.

[49] Viana S de SC , Arantes T , Ribeiro SC , da C . Interventions of the clinical pharmacist in an intermediate care unit for elderly patients. Einstein. 2017;15:283‐288. 29091149 10.1590/S1679-45082017AO3894 PMC5823041

[50] Al‐azzam SI , Shara M , Alzoubi KH , Almahasneh FA , Iflaifel MH . Implementation of clinical pharmacy services at a university hospital in Jordan. Int J Pharm Pract. 2013;21(5):337‐340. 23418903 10.1111/ijpp.12009

[51] Carson GL , Crosby K , Huxall GR , Brahm NC . Acceptance rates for pharmacist‐initiated interventions in long‐term care facilities. Innov Pharm . 2013;4(4). 10.24926/iip.v4i4

[52] Chua SS , Kok LC , Yusof FA , et al. Pharmaceutical care issues identified by pharmacists in patients with diabetes, hypertension or hyperlipidaemia in primary care settings. BMC Health Serv Res. 2012;12(1):1‐10. 23145922 10.1186/1472-6963-12-388 PMC3529120

[53] Kijlstra N , Ridge K , Walser S . Pharmaceutical care: where do we stand‐where should we go?: key concepts in pharmaceutical care, quality assessment of pharmaceutical care in Europe, sources of information: survey Report. European Directorate for the Quality of Medicines & HealthCare (EDQM).; 2009.

[54] Wu JY , Leung WY , Chang S , et al. Effectiveness of telephone counselling by a pharmacist in reducing mortality in patients receiving polypharmacy: randomised controlled trial. BMJ. 2006;333(7567):522. 16916809 10.1136/bmj.38905.447118.2F PMC1562472

[55] Arunmanakul P , Kengkla K , Chaiyasothi T , et al. Effects of pharmacist interventions on heart failure outcomes: a systematic review and meta‐analysis. J Am Coll Clin Pharm. 2021;4(7):871‐882.

