# Supplementary material for: Drug‐related problems among hospitalized hypertensive and heart failure patients and physician acceptance of pharmacists' interventions at a teaching hospital in Ghana
Source: Health Sci Rep. 2022 Aug 24;5(5):e786. doi: 10.1002/hsr2.786 (PMC9401642; doi:10.1002/hsr2.786)
Supplement: Supplementary file 1 — Supporting information. [file HSR2-5-e786-s001.docx]

**Definition of terms**

**Drug related problem:** an event or circumstance involving drug therapy that actually or potentially interferes with desired health outcomes [van Mil, 2017].

**Drug related problems**

**Dose too high:**

The patient has a medical problem that is being treated with too much of the correct medication (toxicity).

**Dose too low:** The patient has a medical problem that is being treated with too little of the correct medication

**Drug interaction:**

The patient has a medical problem that is the result of a drug–drug, drug–food, or drug– laboratory test interaction (manifest interaction) or patient could potentially develop a medical problem that is the result of a drug–drug, drug–food, or drug– laboratory test interaction(potential interaction)

**No drug treatment for existing indication:**

The patient has a medical problem that requires medication therapy (an indication for medication use) but is not receiving a medication for that indication. Eg. Patient has an infection but he/she is not receiving an antibiotic

**Inappropriate drug selection:**

The patient has a medication indication but is taking the wrong medication.

**No clear indication for drug use:**

The patient is taking a medication for no medically valid indication.

**Medication counselling need:**

Patient has defaulted or is non-adherent to medication for his/her current problem or receiving medication with significant potential toxicity, interactions, restrictions, needing detailed information on administration techniques eg: warfarin

**Inappropriate duplication of therapeutic group or active ingredient:**

Patient is receiving drugs with the same active ingredient or different drugs for the same medical problem or indication except where the effects are synergistic.

**Dosage regimen too frequent or not frequent enough:**

Dosing of the drug is less frequent or too frequent except in renal or hepatic impairment or potential drug interaction altering serum levels of the drug

**Duration of treatment too long or too short:**

Duration of treatment too short or long except where an adverse drug reaction has been suffered or lack of therapeutic effect or drug-drug interaction

**Contra-indication for drug use:**

Drug is not to be used in the patient in question

**Inappropriate outcome monitoring**: patient is receiving a drug which requires monitoring of laboratory results, clinical signs and symptoms but has not been instituted or started

Drug under-administered or not administered at all**:** patient has missed at least 1 day of 1 or more of his/her drugs

**Wrong drug taken/administered:**

Patient administered a drug other than the 1 supposed to be administered when a dose is due

**Inappropriate timing of administration and/or dosing intervals:**

Drug administered at before or after scheduled time of administration

**Adverse drug reaction:**

Patient suffers a suspected adverse drug reaction

**Inadequate response:**

patient not achieving improvement in clinical parameters compared to baseline despite optimal drug use (including optimal dose)
